# Effects of deep brain stimulation on prepulse inhibition in obsessive-compulsive disorder

**DOI:** 10.1038/tp.2015.171

**Published:** 2015-11-10

**Authors:** S Kohl, T O J Gruendler, D Huys, E Sildatke, T A Dembek, M Hellmich, M Vorderwulbecke, L Timmermann, S E Ahmari, J Klosterkoetter, F Jessen, V Sturm, V Visser-Vandewalle, J Kuhn

**Affiliations:** 1 Department of Psychiatry and Psychotherapy, University Hospital Cologne, University of Cologne, Cologne, Germany; 2 Faculty of Economics, Otto-von-Guericke-University, Magdeburg, Germany; 3 Center for Behavioral Brain Sciences, Magdeburg, Germany; 4 Department of Neurology, University of Cologne, Cologne, Germany; 5 Institute of Medical Statistics, Informatics, and Epidemiology, University of Cologne, Cologne, Germany; 6 Department of Psychiatry, Center for Neuroscience Program, Center for the Neural Basis of Cognition, University of Pittsburgh, Pittsburgh, PA, USA; 7 Department of Neurosurgery, University of Würzburg, Würzburg, Germany; 8 Department of Stereotactic and Functional Neurosurgery, University of Cologne, Cologne, Germany

## Abstract

Owing to a high response rate, deep brain stimulation (DBS) of the ventral striatal area has been approved for treatment-refractory obsessive-compulsive disorder (tr-OCD). Many basic issues regarding DBS for tr-OCD are still not understood, in particular, the mechanisms of action and the origin of side effects. We measured prepulse inhibition (PPI) in treatment-refractory OCD patients undergoing DBS of the nucleus accumbens (NAcc) and matched controls. As PPI has been used in animal DBS studies, it is highly suitable for translational research. Eight patients receiving DBS, eight patients with pharmacological treatment and eight age-matched healthy controls participated in our study. PPI was measured twice in the DBS group: one session with the stimulator switched on and one session with the stimulator switched off. OCD patients in the pharmacologic group took part in a single session. Controls were tested twice, to ensure stability of data. Statistical analysis revealed significant differences between controls and (1) patients with pharmacological treatment and (2) OCD DBS patients when the stimulation was switched off. Switching the stimulator on led to an increase in PPI at a stimulus-onset asynchrony of 200 ms. There was no significant difference in PPI between OCD patients being stimulated and the control group. This study shows that NAcc-DBS leads to an increase in PPI in tr-OCD patients towards a level seen in healthy controls. Assuming that PPI impairments partially reflect the neurobiological substrates of OCD, our results show that DBS of the NAcc may improve sensorimotor gating via correction of dysfunctional neural substrates. Bearing in mind that PPI is based on a complex and multilayered network, our data confirm that DBS most likely takes effect via network modulation.

## Introduction

Obsessive-compulsive disorder (OCD) is one of the most prevalent and disabling of all psychiatric illnesses.^1^ Core symptoms of OCD are anxiety, provoking recurrent and intrusive thoughts or images (obsessions) and repetitive and ritualistic behaviors (compulsions) that are time consuming in nature (Diagnostic and Statistical Manual of Mental Disorders, Fifth Edition). Cognitive behavioral therapy and selective serotonin reuptake inhibitors are the most effective treatment options available. About 10% of all patients, however, profit neither from pharmacological nor from psychological treatments.^2^ It has been suggested that these treatment-refractory OCD patients (tr-OCD) might benefit from deep brain stimulation (DBS), a stereotactic, neuromodulative procedure that delivers chronic and high frequency stimulation in subcortical regions of the brain.^3^ Since 1999, ~200 otherwise treatment-refractory patients suffering from OCD underwent stereotactic surgery.^4^ Although the most optimal stimulation point is still being determined, DBS for OCD has led to significant symptom reductions in most tr-OCD patients up to this point (for a review, see Kohl *et al.*^5^). Thus, based on the largest data set, and taking into account the severe nature of tr-OCD, the FDA (Food and Drug Administration) approved DBS for the ventral striatal approach (the so-called VC/VS region, which includes the nucleus accumbens (NAcc)) under the Humanitarian Device Exemption (see: http://www.accessdata.fda.gov/scripts/cdrh/cfdocs/cftopic/pma/pma.cfm?num=H050003). CE-marking by European public authorities followed shortly thereafter, despite the fact that the underlying mechanism of action of DBS in OCD is not fully understood.

Similarly, the underlying mechanisms of OCD itself are still unclear. Dysfunctions of cortical–striatal–thalamo–cortico pathways^6, 7^ and a failure of ventral striatum inhibition are two proposed theories of OCD symptom generation.^8^ Recently, Ahmari *et al.*^9^ showed that increasing activity in orbitofrontal cortex–ventromedial striatum connections by means of repetitive optogenetic stimulation induced persistent, perseverative grooming behavior in mice, demonstrating a causal relationship between hyperactivity in a particular cortical–striatal–thalamo–cortico pathway and the generation of OCD-like behavior. Dysfunctions in several neurotransmitter systems have also been related to OCD, including serotonergic, glutamatergic and dopaminergic systems.^10, 11^

The acoustic startle reflex is a primitive and protective whole-body response to a sudden and intense stimulus. It is linked to a pathway that connects the cochlear nuclei, the ventrolateral tegmental nucleus, and the caudal pontine reticular nucleus, which ultimately activates the motor neurons that trigger the startle response.^12^ Prepulse inhibition (PPI) is an operational measure of sensorimotor gating, and is modulated by brain circuits that are involved in OCD and known to be affected by VC/VS DBS. PPI is based on modulation of the acoustic startle reflex. PPI is a robust attenuation of the startle reflex when the startle-eliciting stimulus (the pulse) is preceded by a weaker sensory stimulus (the prepulse). It is known that inputs from the forebrain as well as basal ganglia structures have modulatory effects on the PPI circuit. In particular, NAcc is an important relay nucleus for forebrain structures and limbic regions that together modulate cognition and behavior. It is specifically thought that the NAcc regulates PPI via the dopaminergic system and non-NMDA receptors.^13, 14^ Several studies suggest that PPI is deficient in subgroups of OCD patients.^14^

The aim of the current study was to investigate whether DBS influences PPI. If changes in PPI are observed after basal ganglia function is modulated with DBS, this might shed further light on the mode of action of DBS and its therapeutic effect in OCD. As the PPI circuit and its modulations are well understood, this knowledge if obtained could help to further disentangle the neural mechanisms underlying beneficial effects of NAcc-DBS in OCD. On the basis of the literature, our primary hypothesis is that PPI will be reduced in OCD patients compared with healthy controls. Second, in the DBS patient group, we expect that PPI will increase during stimulation, as compared with the off-stimulation condition.

## Materials and methods

### Participants

**Figure 1:**
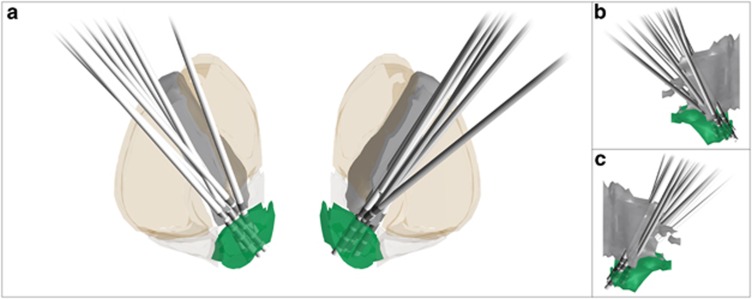
DBS electrodes. Coronal (**a**) and sagittal (**b** and **c**) views of the patient's electrodes in relation to the surrounding brain structures. Electrode coordinates were determined using postoperative CT or stereotactic X-ray. In two patients, the postoperative imaging was not available so planning coordinates are shown. All coordinates were transformed into standardized brain space as shown before.^15^ The nucleus accumbens is shown in green, the internal capsule is shown in gray, and the striatum (including its fundus region) is shown in transparent orange/beige. Three-dimensional brain structures were generated from the ‘Atlas of the Human Brain'.^42^ CT, computed tomography; DBS, deep brain stimulation.

This study has been approved by the Ethics Committee of the Medical Faculty of the University of Cologne. All the participants gave their written informed consent after the nature and possible consequences of the study were explained to him/her. Eight patients (four male) with OCD and DBS of the NAcc (tr-OCD group; see Figure 1), eight OCD patients (seven male) with pharmacological treatment (OCD group) and eight healthy controls with no history of psychiatric illness (four male) participated in the study. Groups were matched for age (for detailed information, see Table 1). All the patients included have been diagnosed with OCD according to the Diagnostic and Statistical Manual of Mental Disorders, Fourth Edition and ICD-10. Stimulation parameters were chosen for best clinical results, based on patients' report. PPI sessions were counter-balanced (four On first) within the group of tr-OCD patients. Though we planned to switch the stimulation device Off for 12 to 24 h, some patients did not tolerate this time frame (see Table 2). To ensure the stability of PPI data over time, we also retested a majority of our control subjects (*n*=6); two controls did not take part in the second session.

### Procedure

The eye blink component of the startle reflex and PPI were measured by electromyogram (EMG) of the orbicularis oculi muscle. EMG was recorded by a commercially available startle system (SR-HLab, San Diego Instruments, San Diego, CA, USA). The procedure was conducted following the guidelines for human startle studies;^16^ for further details regarding the procedure, see also ref. 5. The patients were tested on medication (see Table 3). The background noise was 70 dB(A) sound pressure level (SPL) broadband white noise; acoustic stimuli consisted of bursts of 20 ms white noise with uncontrolled instant rise time. Startle eliciting stimuli were presented at 110 dB(A) SPL, and prepulse stimuli were presented at 80 dB(A) SPL. A test session consisted of three blocks with a total of 60 trials. The first and the third block were identical and consisted of five pulse-alone (PA) trials. The second block was composed of 50 trials, of which 10 were further PA trials, 10 were prepulse alone trials and 30 were prepulse+pulse trials (PP). The PP trials consisted of three groups that differed in their prepulse to pulse stimulus-onset asynchrony (SOA). The paradigm included 10 trials with an SOA of 60 ms (PPI60), 10 trials with an SOA of 120 ms (PPI120) and 10 trials with an SOA of 200 ms (PPI200). All different trial types in the second block were intermixed and presented in a pseudo-randomized order.

### Data acquisition and processing

EMG data were analyzed using a custom written Matlab program (MathWorks, Natick, MA, USA) developed in our clinic to visually inspect and analyze PPI data. Recorded EMG activity was high-pass filtered at 28 Hz and low-pass filtered at 300 Hz using fourth-order Butterworth Filter, and a 50 Hz notch filter was used to reduce power line interference. EMG recorded with DBS ON must be especially pre-processed and visualized owing to the excessive artifact at 130 Hz and harmonics. To minimize the impact of the artifact, a Hampel filter was applied to the signal.^17, 18^ The same filtering was applied to all the data sets. The Hampel filter is a robust outlier detector that will identify values whose absolute difference from the median value is greater than a pre-defined threshold. The filter window was set to 2 Hz between 1 and 290 Hz with a threshold of 5. Owing to short epochs, the outlier detection was not applied to the complex spectrum but to the signal amplitude, taking into account a potential phase shift. The EMG signal was subsequently rectified and smoothed with a moving average at a time constant of 10. By means of visual inspection, any trial featuring excessive noise in the EMG signal or a spontaneous blink in the period immediately preceding the stimulus onset or the minimal response onset were excluded from further analyses. Percentages of trials excluded were all beneath 20% (PA trials 9.21, PPI60 trials: 9.15, PPI120 trials: 16.22, and PPI200 trials: 17.84). Criteria for qualifying the EMG signal as an actual startle response were defined in accordance with guidelines for human startle eye blink EMG studies.^16^ The latency window was set at 20–150 ms after pulse onset, and minimum response amplitude was set at 2 s.d. above baseline, which was defined by the prepulse alone trials. The highest amplitude in the given time window was identified as the response peak. PPI was calculated using the following formula: ((mean PA−mean PP)/mean PA) × 100. PPI values were calculated for the three SOA types separately and for all prepulse trials taken together.

### Statistical analysis

Mean startle magnitudes were calculated by averaging the response magnitude of all included trials. Location differences between distributions were evaluated by linear models adjusted for clustering within patients (sandwich estimator of variance, generalized estimating equations models). A correction for multiple testing was waived so that the statistical power of our experiment is not further diminished. Empirical distributions were checked for normal distribution using the Shapiro–Wilk test with significance level 0.1. Owing to apparent normality, the correlation between symptom severity (YBOCS) and prepulse inhibition was assessed by Pearson's correlation coefficient. Calculations were done using Excel (Microsoft, Redmond, WA, USA), SPSS (IBM, Armonk, NY, USA) and Stata (StataCorp, College Station, TX, USA).

## Results

As the Shapiro–Wilk test yielded *P*-values >0.1 for most data distributions, we decided to apply methods for normal data, which are likely to be more powerful than alternative nonparametric methods. In a multifactorial, linear GEE model of PPI level, both main effects group and PPI condition (*P*<0.001) as well as their interaction (*P*=0.005) were statistically significant. More specifically, the overall PPI value was significantly different between groups (F(3,23)=5.3, *P*=0.007). *Post hoc* comparisons indicated that the tr-OCD group with DBS off (47.3±12.8, *P*=0.001) and the OCD group (46.9±20.2, *P*=0.009) both had significantly decreased PPI compared with the control group (71.3±13.2). Analyzing the different SOAs separately, the linear model revealed a significant effect of group for PPI60 (F(3,23)=4.8, *P*=0.001), PPI120 (F(3,23)=4.3, *P*=0.015), and PPI200 (F(3,23)=7.3, *P*=0.001).

*Post hoc* comparisons of PPI60 indicated significant PPI decreases in all the three groups compared with the control group (tr-OCD group with DBS off (32.1±27.2, *P*=0.015), tr-OCD group with DBS on (40.4±22.8, *P*=0.042); OCD group (33.7±17.3, *P*=0.003); control group (61.3±15.1)).

*Post hoc* comparison of PPI120 similarly revealed significant PPI decreases in all the three groups compared with the control group (tr-OCD group with DBS off (70.9±12.2, *P*=0.016); tr-OCD group with DBS on (65.2±19.3, *P*=0.014); OCD group (61.8±24.8, *P*=0.018); control group (86.5±11.5)).

**Figure 2:**
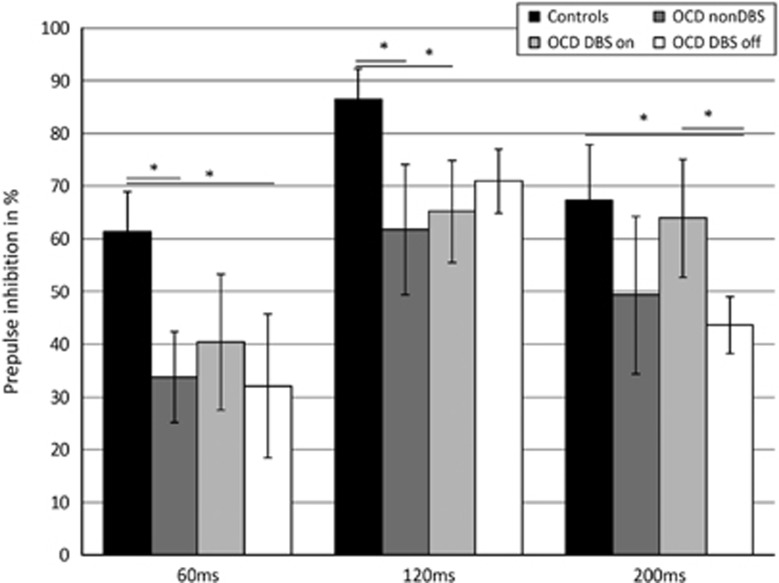
Prepulse inhibition. Prepulse inhibition (PPI) in percent; 60 ms/120 ms/200 ms: stimulus-onset asynchrony (SOA) between prepulse and pulse of 60 ms, 120 ms and 200 ms, respectively. All: PPI calculated including all SOA conditions. *Significant difference between groups according to linear model and *post hoc* comparison (*P*<0.05). DBS, deep brain stimulation; OCD, obsessive-compulsive disorder.

Finally, *post hoc* comparison of PPI200 revealed significant decreases in the tr-OCD group with DBS off (43.6±10.7) compared with both the control group (67.3±21.2, *P*=0.010) and the tr-OCD group with DBS on (63.9±22.4, *P*=0.003; see Figure 2).

We did not find any significant differences between the first (71.3±13.2) and second (70.3±17, *P*=0.901) test session of the controls, which indicates that the startle paradigm produces longitudinally stable data over 6 months. We further did not find any significant differences between groups on the mean startle magnitude (F(3,23)=2.3, *P*=0.101).

### Correlation

In OCD patients, we found a negative correlation of −0.60 (*P*<0.001) between symptom severity measured with the YBOCS and PPI. Thus, higher symptom severity was associated with reduced PPI.

## Discussion

The aim of this study was to gain insight into the acute network effects of NAcc-DBS in patients with tr-OCD. We used PPI as a neurophysiological paradigm with well-understood neural circuitry, based on a network of midbrain colliculi that are modulated by NAcc.^19^ PPI is a paradigm of high translational relevance, as it is disrupted in many psychiatric disorders, and PPI disruptions are even considered by some authors to be an endophenotype in schizophrenia.^20^ Furthermore, it is present in animal models, and has been used for translational psychiatric studies. We expected that dysfunction of PPI would be present in severe OCD patients, even though previous studies have not been entirely consistent. In line with our initial prediction, we found that patients had significantly decreased PPI compared with healthy controls in the between-group comparison. We also found a correlation between symptom severity and OCD, that is, the more severe the symptoms, the more pronounced the presumptive neurobiological deficits resulting in greater PPI deficits.

PPI impairments have been reported in different psychiatric disorders that have a loss of cognitive, motor or sensory gating in common.^21^ Theories of sensory gating^22^ and the protection of pre-attentive processing^23^ suggest that filtering the input enables uninterrupted processing of information. As in everyday life gating occurs tonically, improved PPI, as a measure of gating, may lead to more continuous thought or action,^21^ which in turn might lead to clinical improvement in OCD patients.

To date, four studies using OCD patients have been published, all of which used SOAs of 120ms, and none of which investigated different intertrial intervals. In 1993, Swerdlow *et al.*^24^ published a preliminary assessment of PPI in patients suffering from OCD, including 11 patients and 13 controls. The results suggested a possible impairment of sensorimotor gating in patients with OCD, only in trials with a prepulse of 4 dB (A) above the background noise. However, no overall significant difference was reported. Results of Hoenig *et al.*^25^ were similar. Although statistical analysis revealed a significant difference, *post hoc* analyses showed that only in one trial type, 16 dB (A) prepulse trials, PPI was reduced in OCD patients compared with controls. De Leeuw *et al.*^26^ reported no difference between drug-naive patients and controls. In contrast, Ahmari *et al.*^27^ found that PPI was deficient for all prepulse intensities in unmedicated OCD patients (4, 8, 16 dB above background white noise of 70 dB; 22 OCD patients and 22 matched controls). Furthermore, they found that OCD patients with a history of tic disorder were more likely to have reduced PPI values.

The within-group comparison of NAcc-DBS showed a significant difference between the stimulation on and stimulation off condition in the 200 ms SOA trial type. The PPI process varies depending on the SOA duration, shorter SOAs reflect more pre-attentive and automatic processing, whereas longer SOAs are assumed to include attentive and controlled processes.^28^ From our study, one might conclude that DBS affects attentive mechanisms stronger than the pre-attentive ones. But a trend can also be observed in the scores of the 60 ms SOA trial type, showing that tr-OCD patients exhibit better inhibition when stimulation is on compared to off. Replication of the results will have to show whether DBS affects both pre-attentive and attentive aspects of PPI, or only selective SOA conditions. Comparing the different stimulation parameters, we did not find any significant effect of stimulation settings on PPI.

Denys and colleagues recently showed that NAcc-DBS modulates NAcc activity and frontostriatal connectivity, and thereby reverses disease-related hyperactivity of the cortical–striatal–thalamo–cortico loop.^29^ Our results further underline the neuromodulatory potency of DBS, as it is known that PPI disruptions are relatively stable,^30^ as evidenced by our control data demonstrating stability over two sessions separated in time. Furthermore, we showed that DBS of the NAcc has no negative influence on PPI, which has been observed to be the case when used in healthy rats.

What are putative explanations for these effects of DBS on PPI? Dopamine, glutamate, serotonin and acetylcholine are all involved in the neurochemical regulation of PPI as shown via systematic drug studies. Although OCD is primarily associated with dysfunctions in the serotonergic system, recent observations suggest that dopaminergic activity may also have a decisive role, at least in a subgroup of OCD patients. Our patients, who are treatment resistant to selective serotonin reuptake inhibitors, might belong to this specific subgroup. The relation of dopaminergic activity and PPI is complex. Translational studies suggest that disrupted PPI is related to hypodopaminergic function in the prefrontal cortex and/or to hyperdopaminergic subcortical regions, such as the striatum. Lesion studies as well as pharmacological interventions support this hypothesis. Different lines of evidence support the hypothesis of a hyperactive dopamine system in OCD. First, dopamine antagonists have been found to be effective as augmentation to selective serotonin reuptake inhibitors in some groups of OCD patients.^31, 32^ Second, stimulant drugs elevating the dopamine level may induce OCD-like behavior.^33^ Third, molecular imaging studies indicate a reduction in dopamine receptor binding in patients with OCD compared with controls.^34, 35^ Reduced binding may either indicate reduced receptor density or increased endogenous dopamine. The efficacy of DBS might therefore be explained via normalization of dopaminergic homeostasis. A similar mechanism has already been demonstrated in Tourette patients during thalamic stimulation.^36^ In addition, this hypothesis is supported by recently published animal data. It has been shown that DBS of the NAcc in rats could reverse ketamine-induced PPI deficits, but did not influence PPI in healthy animals. The authors suggest that NAcc stimulation acts upon the dopaminergic pathway from the ventral tegmental area to the NAcc, and influences PPI via its ventral pallidum output.^37^ Recent evidence from human^38^ and translational studies^39^ suggest that NAcc-DBS further increases dopamine, which would be contradictory to its clinically beneficial effect and the before-mentioned clinical observations of dopamine in OCD. Another possible explanation might thus be an alteration of oscillations in the stimulated networks. Analysis has shown that DBS of the NAcc has distinct influences on the network,^40^ and indirectly that NAcc-DBS affects pathological oscillatory connectivity in humans.^29^ Pathological gamma oscillations can also provoke disruptions in PPI.^41^

This study has several limitations. First, female participants were not tested in the same hormonal status. Furthermore, the number of smokers is not the same in each group. Also, the overall sample size is small, owing to the unique method of DBS. Finally, the off interval for stimulation differed between patients because some did not agree to switch the stimulator off overnight. Participants were tested on their regular medications, which partly differed between on and off test sessions. This can be a potential confounding factor, especially in the case of anti-dopaminergic agents. We decided not to present any corrected *P*-values so that the sensitivity (or power) of our paradigm is not lost; however, the reader may easily apply the correction themselves (that is, by multiplication of the *P*-values with 6 (per PPI condition) or 24 (=4 × 6) overall). Nevertheless, we recommend validating our results in an independent and bigger sample of patients as the present study serves investigative purposes and needs replication.

In conclusion, we found that patients with OCD exhibit decreased PPI compared with controls. A strong negative correlation between symptom severity and PPI further indicated stronger PPI deficits in patients with more severe OCD. DBS improved PPI, although this effect was only significant in the 200 ms SOA condition, which might be an indication that DBS modulates the attentive more than the pre-attentive processes of PPI. It is possible that network modulation by DBS causes a normalization of the PPI circuit, and may serve as an indication of the neurobiological basis of DBS efficacy.

## Figures and Tables

**Table 1:** Demographic and clinical information

	*tr-OCD (DBS)*		*OCD (non-DBS)*		*Controls*	
	*Mean*	*s.d.*	*Mean*	*s.d.*	*Mean*	*s.d.*
Age (years)	40.8	12.1	36.4	16.3	41.0	9.1
YBOCS	30.8	8.7	23.5	8.6	NA	NA
YBOCS On	24.0	8.3	NA	NA	NA	NA
YoD	22.3	9.1	12.5	12.9	NA	NA
Smoking	4 Smokers		1 Smoker		3 Smokers	

Abbreviations: DBS, deep brain stimulation; NA, not applicable; OCD, obsessive-compulsive disorder; tr-OCD, treatment-refractory OCD; YBOCS, Yale Brown Obsessive-Compulsive scale; YBOCS On, YBOCS score when tested in stimulation on condition; YoD, years since OCD diagnosis.

**Table 2:** Stimulation parameters

*Patient*	*Time since surgery*	*Amplitude (V)*	*Frequency (Hz)*	*Pulse width (μs)*	*Active poles*	*Time stim. Off*
tr-OCD 01	36	1.5	130	90	0,1,2,8,9,10	17
tr-OCD 02	72	5.5	120	150	2,3,10,11	3
tr-OCD 03	38	5.0	130	150	1,3,9,10	13
tr-OCD 04	25	3.8	130	90	2,3,10,11	10
tr-OCD 05	13	4.5	130	150	1,2,9,10	24
tr-OCD 06	3	7.0	130	120	2,3,10,11	10
tr-OCD 07	12	6.0	130	150	0,1,8,9	19
tr-OCD 08	12	5.5	130	120	0,1,8,9	10

Abbreviation: tr-OCD, treatment-refractory obsessive-compulsive disorder.

Time since surgery indicates time since stereotactic implantation in months. Active poles indicates active monopolar contacts. Time stim. Off indicates time span in hours the DBS stimulator was set Off according to the patient's agreement.

**Table 3:** Medication

*OCD patient*	*Medication*
OCD 01	Fluoxetine
OCD 02	Citalopram, L-thyroxin
OCD 03	NA
OCD 04	NA
OCD 05	Citalopram, quetiapine, memantin, L-thyroxin
OCD 06	Citalopram, valproate, quetiapine, promethazine
OCD 07	Lorazepam, chlorprothixene, mirtazapine, ramipril, bisoprolol, sertralin, L-thyroxin, akineton, paliperidone
OCD 08	Amitriptyline, diazepam, valsartan, amlodipine, metoprolol, ASS, pantoprazole, trazodone

*DBS patient*	*Medication DBS On*	*Medication DBS Off*
tr-OCD 01	Fluoxetine, lavender essential oil, Cerazette	Aripiprazole, fluoxetine
tr-OCD 02	Diazepam, aripiprazole, clomipramine, chlorprothixene, thyroxin	Diazepam, aripiprazole, clomipramine, chlorprothixene, thyroxin
tr-OCD 03	Duloxetine, quetiapine, bisoprolol	Duloxetine, quetiapine, bisoprolol
tr-OCD 04	Citalopram, olanzapine, venlafaxine	Citalopram, olanzapine
tr-OCD 05	Quetiapine, ibuprofen	Eucreas, citalopram, asenapine, ramipril, quetiapine, zopiclone
tr-OCD 06	Medikinet adult	Paroxetine, Medikinet adult
tr-OCD 07	Fluoxetine, pregabalin	Fluoxetine, pregabalin
tr-OCD 08	Sertraline, clomipramine, agomelatine, aripiprazole	NA

Abbreviations: DBS, deep brain stimulation; NA, not applicable; OCD, obsessive-compulsive disorder; tr-OCD, treatment-refractory OCD.

Psychopharmacological medication on the day of testing for each patient, for DBS patients medications for test sessions On and Off, respectively.
